# Supplementary material for: Is indoor environment a risk factor of building-related symptoms?
Source: PLoS One. 2023 Jan 25;18(1):e0279757. doi: 10.1371/journal.pone.0279757 (PMC9876365; doi:10.1371/journal.pone.0279757)
Supplement: S1 Table — This table is the substances identified and analyzed in Case1. (DOCX) [file pone.0279757.s001.docx]

**Supplementary Tables1.** **VOCs and OAVs (Case 1).**

| **Case 1 ΣVOC level < 400 µg/m^3^** | | | VOC　(μg/m^3^) | | OAV |
| --- | --- | --- | --- | --- | --- |
|  | Mid. | Frequency | Mid. | Frequency |  |
| α-Pinene | 92.50 | 100.00% | 0.92 | 34.04% |  |
| Dichloromethane | 52.20 | 100.00% | N.D. |  |  |
| 3-Carene | 23.38 | 100.00% | N.D. |  |  |
| Formaldehyde | 22.10 | 100.00% | 0.04 | 0.00% |  |
| Tridecane (Texanol) | 15.80 | 100.00% | N.D. |  |  |
| Acetone | 8.40 | 100.00% | N.D. |  |  |
| Acetaldehyde | 9.60 | 100.00% | 3.55 | 100.00% |  |
| Toluene | 4.00 | 100.00% | N.D. |  |  |
| Limonene | 5.80 | 100.00% | 0.03 | 0.00% |  |
| Ethylacetate | 3.60 | 100.00% | N.D. |  |  |
| 2-Butanone　(Methyl ethyl ketone) | 3.40 | 100.00% | N.D. |  |  |
| β-Pinene | 4.80 | 100.00% | 0.03 | 0.00% |  |
| Nonanal (n-Nonylaldehyde) | 4.00 | 100.00% | 2.02 | 100.00% |  |
| n-Butylacetate | 4.60 | 100.00% | 0.06 | 0.00% |  |
| n-Butanol | 4.50 | 100.00% | 0.04 | 0.00% |  |
| 2-Ethyl-1-hexanol (Isooctanol) | 2.50 | 100.00% | 0.05 | 0.00% |  |
| Hexadecane | 1.30 | 91.49% | N.D. |  |  |
| Decanal (n-Decylaldehyde) | 2.30 | 65.96% | N.D. |  |  |
| 2-Propanol | 1.40 | 61.70% | N.D. |  |  |
| Ethylbenzene | 1.70 | 44.68% | N.D. |  |  |
| TXIB | 1.40 | 42.55% | N.D. |  |  |
| Phenol | 12.20 | 40.43% | N.D. |  |  |
| Xylene | 1.60 | 27.66% | N.D. |  |  |
| 1-Propanol | 2.40 | 17.02% | N.D. |  |  |
| n-Decane | 1.00 | 8.51% | N.D. |  |  |
| Tetradecane | 1.00 | 8.51% | N.D. |  |  |
| n-Hexane | 1.20 | 6.38% | N.D. |  |  |
| Benzene | N.D. |  |  |  |  |
| Bromodichloromethane | N.D. |  |  |  |  |
| Chloroform | N.D. |  |  |  |  |
| Dibromochloromethane | N.D. |  |  |  |  |
| Dodecane | N.D. |  |  |  |  |
| Ethanol | N.D. |  |  |  |  |
| Isooctane | N.D. |  |  |  |  |
| m-Ethyltoluene | N.D. |  |  |  |  |
| n-Heptane | N.D. |  |  |  |  |
| n-Octan | N.D. |  |  |  |  |
| Nonane | N.D. |  |  |  |  |
| o-Ethyltoluene | N.D. |  |  |  |  |
| p-Dichlorobenzene | N.D. |  |  |  |  |
| Pentadecane | N.D. |  |  |  |  |
| p-Ethyltoluene | N.D. |  |  |  |  |
| Styrene | N.D. |  |  |  |  |
| Tetrachloroethylene | N.D. |  |  |  |  |
| Trichloroethylene | N.D. |  |  |  |  |
| Tridecane | N.D. |  |  |  |  |
| Undecane | N.D. |  |  |  |  |
| 4-Methyl-2-pentanone | N.D. |  |  |  |  |
| 2,4-Dimethylpentane | N.D. |  |  |  |  |
| 1,2-Dichloropropane | N.D. |  |  |  |  |
| 1,2-Dichloroethane | N.D. |  |  |  |  |
| 1,2,4,5-Tetramethylbenzene | N.D. |  |  |  |  |
| 1,2,3-Trimethylbenzene | N.D. |  |  |  |  |
| 1,3,5-Trimethylbenzene | N.D. |  |  |  |  |
| 1,2,4-Trimethylbenzene | N.D. |  |  |  |  |
| N.D.: Not detected,  TXIB: 2,2,4-Trimethyl-1,3-pentanediol-diisobutyrate,  VOC: volatile organic compound,  OAV: odor activity value |  |  |  |  |  |
