# Supplementary material for: Is indoor environment a risk factor of building-related symptoms?
Source: PLoS One. 2023 Jan 25;18(1):e0279757. doi: 10.1371/journal.pone.0279757 (PMC9876365; doi:10.1371/journal.pone.0279757)
Supplement: S2 Table — This table is the substances identified and analyzed in Case2. (DOCX) [file pone.0279757.s002.docx]

**S2 Table** **VOCs and OAVs (Case 2).**

| **Case 2 ΣVOC level > 400 µg/m^3^** | VOC　(μg/m^3^) | | OAV | |
| --- | --- | --- | --- | --- |
|  | Mid. | Frequency | Mid. | Frequency |
| Dichloromethane | 593.93 | 100.00% | N.D. |  |
| α-Pinene | 514.25 | 100.00% | 5.13 | 100.00% |
| 3-Carene | 148.63 | 100.00% | N.D. |  |
| Acetone | 65.72 | 100.00% | N.D. |  |
| Acetaldehyde | 31.52 | 100.00% | 11.66 | 100.00% |
| Formaldehyde | 38.31 | 100.00% | 0.06 | 0.00% |
| Ethylacetate | 31.74 | 100.00% | 0.01 | 0.00% |
| β-Pinene | 27.43 | 100.00% | 0.15 | 0.00% |
| Limonene | 26.10 | 100.00% | 0.12 | 0.00% |
| n-Butanol | 16.29 | 100.00% | 0.14 | 2.56% |
| Toluene | 14.30 | 100.00% | 0.01 | 0.00% |
| Ethylbenzene | 8.01 | 100.00% | 0.01 | 0.00% |
| 2-Ethyl-1-hexanol (Isooctanol) | 111.70 | 98.72% | 2.25 | 69.23% |
| 2-Butanone　(Methyl ethyl ketone) | 16.39 | 98.72% | 0.01 | 0.00% |
| Nonanal (n-Nonylaldehyde) | 8.58 | 96.15% | 4.31 | 96.15% |
| n-Butylacetate | 28.42 | 94.87% | 0.36 | 15.38% |
| n-Decane | 5.55 | 91.03% | N.D. |  |
| Tridecane　(Texanol) | 778.91 | 87.18% | 0.01 | 0.00% |
| 1,2,4-Trimethylbenzene | 2.95 | 85.90% | N.D. |  |
| 2-Propanol | 8.33 | 85.90% | N.D. |  |
| Xylene | 4.34 | 85.90% | 0.01 | 0.00% |
| Tetradecane | 2.91 | 84.62% | N.D. |  |
| Styrene | 13.24 | 82.05% | 0.08 | 0.00% |
| Hexadecane | 2.99 | 79.49% | N.D. |  |
| n-Hexane | 4.54 | 78.21% | N.D. |  |
| p-Dichlorobenzene | 2.03 | 78.21% | N.D. |  |
| m-Ethyltoluene | 2.58 | 76.92% | N.D. |  |
| 4-Methyl-2-pentanone | 4.20 | 76.92% | N.D. |  |
| n-Heptane | 29.92 | 75.64% | 0.01 | 0.00% |
| Benzene | 20.68 | 73.08% | N.D. |  |
| 1-Propanol | 38.17 | 67.95% | 0.10 | 0.00% |
| p-Ethyltoluene | 1.86 | 60.26% | N.D. |  |
| Nonane | 8.02 | 55.13% | N.D. |  |
| o-Ethyltoluene | 1.97 | 47.44% | N.D. |  |
| 2,4-Dimethylpentane | 2.97 | 46.15% | N.D. |  |
| 1,3,5-Trimethylbenzene | 2.02 | 37.18% | N.D. |  |
| Dodecane | 2.34 | 35.90% | N.D. |  |
| Trichloroethylene | 1.30 | 24.36% | N.D. |  |
| Decanal (n-Decylaldehyde) | 1.63 | 23.08% | N.D. |  |
| Undecane | 6.89 | 19.23% | N.D. |  |
| 1,2,3-Trimethylbenzene | 9.35 | 17.95% | N.D. |  |
| n-Octan | 1.88 | 14.10% | N.D. |  |
| TXIB | 1.70 | 11.54% | N.D. |  |
| 1,2,4,5-Tetramethylbenzene | 18.41 | 10.26% | N.D. |  |
| 1,2-Dichloropropane | 0.92 | 2.56% | N.D. |  |
| Dibromochloromethane | 0.44 | 2.56% | N.D. |  |
| Pentadecane | 3.33 | 1.28% | N.D. |  |
| Bromodichloromethane | N.D. |  |  |  |
| Chloroform | N.D. |  |  |  |
| Ethanol | N.D. |  |  |  |
| Isooctane | N.D. |  |  |  |
| Phenol | N.D. |  |  |  |
| Tetrachloroethylene | N.D. |  |  |  |
| Tridecane | N.D. |  |  |  |
| 1,2-Dichloroethane | N.D. |  |  |  |
| N.D.: Not detected,  TXIB: 2,2,4-Trimethyl-1,3-pentanediol-diisobutyrate,  VOC: volatile organic compound,  OAV: odor activity value |  |  |  |  |
